# Combination of variations in inflammation- and endoplasmic reticulum-associated genes as putative biomarker for bevacizumab response in *KRAS* wild-type colorectal cancer

**DOI:** 10.1038/s41598-020-65869-2

**Published:** 2020-06-17

**Authors:** Ana Barat, Dominiek Smeets, Bruce Moran, Wu Zhang, Shu Cao, Sudipto Das, Rut Klinger, Johannes Betge, Verena Murphy, Orna Bacon, Elaine W. Kay, Nicole C. T. Van Grieken, Henk M. W. Verheul, Timo Gaiser, Nadine Schulte, Matthias P. Ebert, Bozena Fender, Bryan T. Hennessy, Deborah A. McNamara, Darran O’Connor, William M. Gallagher, Chiara Cremolini, Fotios Loupakis, Aparna Parikh, Christoph Mancao, Bauke Ylstra, Diether Lambrechts, Heinz-Josef Lenz, Annette T. Byrne, Jochen H. M. Prehn

**Affiliations:** 1 Centre for Systems Medicine and Department of Physiology & Medical Physics, Royal College of Surgeons in Ireland, Dublin, Ireland; 2 VIB Vesalius Research Center, KU Leuven, Leuven, Belgium; 3 UCD Conway Institute, University College Dublin, Dublin, Ireland; 4 USC Norris Comprehensive Cancer Center, Los Angeles, USA; 5 Molecular and Cellular Therapeutics, Royal College of Surgeons in Ireland, Dublin, Ireland; 6 UCD, School of Biomolecular and Biomedical Science, Dublin, Ireland; 7 Department of Medicine II, University Hospital Mannheim, Medical Faculty Mannheim, Heidelberg University, Mannheim, Germany; 8 German Cancer Research Center (DKFZ), Division Signaling and Functional Genomics, Heidelberg, Germany; 9 Cancer Trials Ireland, Dublin, Ireland; 10 Department of Pathology, Beaumont Hospital, Dublin, Ireland; 11 Department of Pathology, VU University Medical Center, Amsterdam, The Netherlands; 12 Department of Medical Oncology, VU University Medical Center, Amsterdam, The Netherlands; 13 Institute of Pathology, University Hospital Mannheim, Medical Faculty Mannheim, Heidelberg University, Mannheim, Germany; 14 OncoMark Ltd., NovaUCD, Belfield Innovation Park, Dublin, Ireland; 15 Department of Medical Oncology, Beaumont Hospital, Dublin, Ireland; 16 Department of Surgery, Beaumont Hospital, Dublin, Ireland; 17 Unit of Medical Oncology 2, Department of Translational Research and New Technologies in Medicine and Surgery, Azienda Ospedaliera Universitaria Pisana, Pisa, Italy; 18 Oncology Unit, Istituto Oncologico Veneto, IOV-IRCCS, Padua, Italy; 19 Division of Hematology and Oncology, Massachusetts General Hospital, Boston, USA; 20 Oncology Biomarker Development, Genentech Inc., San Francisco, USA

**Keywords:** Cancer epidemiology, Colorectal cancer

## Abstract

Chemotherapy combined with the angiogenesis inhibitor bevacizumab (BVZ) is approved as a first-line treatment in metastatic colorectal cancer (mCRC). Limited clinical benefit underpins the need for improved understanding of resistance mechanisms and the elucidation of novel predictive biomarkers. We assessed germline single-nucleotide polymorphisms (SNPs) in 180 mCRC patients (Angiopredict [APD] cohort) treated with combined BVZ + chemotherapy and investigated previously reported predictive SNPs. We further employed a machine learning approach to identify novel associations. In the APD cohort *IL8* rs4073 any A carriers, compared to TT carriers, were associated with worse progression-free survival (PFS) (HR = 1.51, 95% CI:1.03–2.22, *p-value* = 0.037) and *TBK1* rs7486100 TT carriers, compared to any A carriers, were associated with worse PFS in *KRAS* wild-type (wt) patients (HR = 1.94, 95% CI:1.04–3.61, *p-value* = *0.037*), replicating previous findings. Machine learning identified novel associations in genes encoding the inflammasome protein *NLRP1* and the ER protein Sarcalumenin (SRL). A negative association between PFS and carriers of any A at *NLRP1* rs12150220 and AA for *SRL* rs13334970 in APD *KRAS* wild-type patients (HR = 4.44, 95% CI:1.23–16.13, *p-value *= 0.005), which validated in two independent clinical cohorts involving BVZ, MAVERICC and TRIBE. Our findings highlight a key role for inflammation and ER signalling underpinning BVZ + chemotherapy responsiveness.

## Introduction

Colorectal cancer (CRC) is the third most commonly diagnosed cancer in both men and women and is associated with high mortality and morbidity^1^. Almost half of patients diagnosed with CRC develop metastatic disease (mCRC). Outcomes of mCRC have improved with introduction of targeted agents such as monoclonal antibodies directed at the epidermal growth factor receptor (EGFR) and the vascular endothelial growth factor (VEGF). Current treatments for mCRC includes 5-fluorouracil-based treatment regimens (XELOX, FOLFOX, FOLFIRI, FOLFOXIRI), with the addition of monoclonal antibodies against EGFR (cetuximab or panitumumab; indicated for *RAS* and *BRAF* wild-type tumours) or VEGF (bevacizumab (BVZ), either as first, second, or third line therapy. Results from phase III clinical trials have demonstrated that the addition of BVZ to cytotoxic chemotherapy improves response rate as well as prolongs PFS and overall survival (OS) of mCRC patients^2,3^. Nevertheless, only a subset of patients respond and overall clinical benefit is limited. Moreover, BVZ therapy is associated with a significant side effect profile including hypertension, renal toxicity, bleeding, wound-healing complications, gastrointestinal perforations, and thromboembolic events^4^.

Resistance to bevacizumab has been linked to several signaling pathways, such as VEGF (Vascular Endothelial Growth factor)-associated alterations in angiogenesis, non-VEGF compensatory mechanisms of blood vessel formation, and stromal cell interactions^5^. Hypothesizing that genetically stable endothelial cells or stromal cells play an essential role in anti-VEGF responsiveness, we and others^6–14^ have assessed single-nucleotide polymorphisms (SNPs) in several candidate genes for their association with clinical responses. However validation of identified SNPs in independent cohorts often proved to be difficult. For example, among ten previously identified candidate SNPs^6,15^, association with treatment outcome could be validated in a larger mCRC cohort only for one SNP in *VEGFR2* rs12505758^9^, with C variants for *VEGFR2* rs12505758 being associated with shorter PFS and OS. Another predictive SNP that was replicated in several cohorts was rs4073 in the *IL8* promoter. Presence of the minor allele A in rs4073 correlates with increased IL-8 production after stimulation of whole blood with lipopolysaccharide^16^, and was associated with poor response to BVZ^10,11^. More recently, it has been reported that AA carriers for rs8602 on *MKNK1*, a gene that upregulates angiogenic factors, had worse PFS in *KRAS* wt patients^13^, and AA carriers for rs4588 on *GC*, a vitamin D-binding protein, had shorter OS^14^ in two independent cohorts of patients treated with BVZ + chemotherapy.

Notwithstanding these studies, identifying and validating predictive biomarkers for BVZ remains an urgent clinical need. To this end we have performed exome sequencing to assess germline SNPs in a retrospective cohort of 180 mCRC patients treated with BVZ + chemotherapy regimens as part of the ANGIOPREDICT (APD) study^17–19^. The patient characteristics of the APD cohort are given in Table 1. We have assessed previously reported predictive germline SNPs (*VEGFA* rs833061; *VEGFR1* rs9513070, rs7993418, rs9582036; *VEGFR2* rs1531289, rs2305948; rs11133360; *IL8* rs4073; *CCL2* rs4586; *TBK1* rs7486100) for their association with BVZ + chemotherapy responsiveness, and have identified novel associations using machine learning (ML). ML has emerged as a powerful computational tool which utilizes a variety of analytical algorithms to iteratively learn from data points. It has been successfully employed to investigate complex genomic and proteomic data sets to identify prognostic signatures^20,21^. In the present study, we have employed repeated cross-validated Cox penalized regression as a ML approach to select SNPs as candidate biomarkers to predict therapy outcome, and validated novel identified SNPs in two independent clinical cohorts.

**Table 1:** Patient characteristics in the APD, MAVERICC and TRIBE cohorts.

Characteristics	Total n = 558	ANGIOPREDICT (chemo BEV)	MAVERICC (FOLFIRI BEV)	TRIBE (FOLFIRI BEV)	Chi-square test p-value
**Sex**					0.78
Male	343	108(60%)	103(63%)	132(61%)	
Female	215	72(40%)	60(37%)	83(39%)	
**Age**					<0.001
≤65	334	77(43%)	101(62%)	156(73%)	
>65	220	99(55%)	62(38%)	59(27%)	
Unknown		4(2%)			
**Grade**					NA
1,2		151(84%)	NA	NA	
3,4		17(10%)	NA	NA	
Unknown		12(6%)	NA	NA	
**T-staging**					NA
2,3		138(77%)	NA	NA	
4		37(20%)	NA	NA	
Unknown		5(3%)	NA	NA	
**N-staging**					NA
0		46(25%)	NA	NA	
1		63(35%)	NA	NA	
2		59(33%)	NA	NA	
X		6(7%)			
**Center**					NA
CAIRO		105(58%)	NA	NA	
RCSI		21(12%)	NA	NA	
UHEI		41(23%)	NA	NA	
VUMC		13(7%)	NA	NA	
**Chemotherapy backbone**					0.042
FP-based	555	177(98%)	163(100%)	215(100%)	
Non-FP	3	3(2%)	0(0%)	0(0%)	
**BVZ Therapy Line**					<0.001
First	539	161(89%)	163(100%)	215(100%)	
Second	12	12(6%)	0(0%)	0(0%)	
Higher	7	7(4%)	0(0%)	0(0%)	
**Primary tumor site**					NA
Colon		128(71%)	NA	NA	
Rectum		52(29%)	NA	NA	
**Primary tumor site**					0.005
Right-sided	120	NA	67(41%)	53(25%)	
Left-sided	243	NA	96(59%)	147(68%)	
Unknown	15	NA	0(0%)	15(7%)	
**Performance status**					<0.001
ECOG 0	274	NA	97(60%)	177(82%)	
ECOG 1	103	NA	66(40%)	37(17%)	
Unknown	1	NA	0(0%)	1(1%)	
**Number of metastases**					<0.001
<2	198	NA	106(65%)	92(43%)	
≥2	180	NA	57(35%)	123(57%)	
**Liver limited disease**					NA
No	150	NA	NA	150(70%)	
Yes	65	NA	NA	65(30%)	
**Primary tumor resected**					<0.001
No	233	NA	153(94%)	80(37%)	
Yes	145	NA	10(6%)	135(63%)	
**Adjuvant chemotherapy**					1
No	331	NA	143(88%)	188(87%)	
Yes	47	NA	20(12%)	27(13%)	
***KRAS*** **status**					0.10
Wild-type		87(48%)	87(54%)	88(41%)	
Mutant		60(33%)	56(34%)	90(42%)	
Unknown		33(18%)	20(12%)	37(17%)	
***BRAF*** **status**					0.14
Wild-type		153(85%)	NA	168(78%)	
Mutant		18(10%)	NA	10(5%)	
Unknown	46	9(5%)	NA	37(17%)	

Footnote for Table 1: FP, fluoropyrimidine; UHEI, University Hospital Mannheim, Heidelberg University, Mannheim, Germany; VUMC, VU Medical Center Amsterdam, Amsterdam, Netherlands; RCSI, Royal College of Surgeons in Ireland, Beaumont Hospital, Dublin, Ireland; CAIRO, Subgroup of patients from CAIRO2 trial, recruited at centers in the Netherlands, that commenced treatment with BVZ between 12.08.2005 and 06.08.2008^41^.

## Results

### Analysis of genetic variation candidates from previous studies

A number of SNP candidates have been proposed for association with response to BVZ + chemotherapy. We selected SNPs based on their biological plausibility, or replication in more than one study (Table 2) and for both dominant and recessive genetic models, we used Cox proportional hazards model adjusting for all available co-variates to test their association with clinical outcome in APD.

**Table 2:** Replication for SNPs previously associated with outcome in cancer patients treated with BVZ + chemotherapy in the APD cohort. HRs, the respective 95% confidence intervals and the across-genotype Wald test p-values are presented for multivariable Cox proportional hazard regression models adjusted for available (see Methods) covariates. When a dominant (d) or a recessive (r) model were most significant, the respective hazard ratios are given under the HR heading.

SNP	HUGO	Major Allele/Minor Allele	Association with PFS in all APD			Association with OS in all APD			Previous findings
			HR	CI	Wald test p-value	HR	CI	Wald test p-val	
			*Agreement for the TT genotypes:*						**Any T carriers have worse OS and PFS in** ***KRAS*** **wt CRC patients treated with first-line FOLFIRI + BVZ**^26^**. Association not found for CRC patients who did not receive BVZ**^26^.
**rs7486100**	***TBK1***	A/T	**1.47 r**	**0.97–2.23**	**0.067**	**1.63 r**	**1–2.38**	**0.034**	
			In APD wild-type *KRAS* (87 patients):						
			**1.94 r**	**1.04–3.61**	**0.037**	**1.86 r**	**0.94–3.68**	**0.074**	
**rs4586**	***CCL2***	T/C	0.81 d	0.56–1.16	0.24	0.86 d	0.59–1.25	0.45	**C carriers have better PFS in** ***KRAS*** **mutant patients treated with first-line FOLFIRI + BVZ**^26^.
			In APD *KRAS* mutant (60 patients):						
			0.82 d	0.47–1.45	0.50	0.92 d	0.5–1.67	0.77	
**rs4073**	***IL8(CXCL8)***	T/A	**1.51 d**	**1.03–2.22**	**0.037**	1.01 d	0.66–1.54	0.96	**TT carriers have better OR**^10^**, PFS**^24^ **in CRC and Response Rate in ovarian cancer**^25^**. Also, AA associated with poor outcome**^11^ **in CRC. Treatment in all cases: BVZ + chemotherapy**.
**rs1531289**	***VEGFR2/KDR***	C/T	0.77 d	0.54–1.09	0.14	0.90 d	0.62–1.31	0.60	**CC (GG) carriers have better PFS in advanced CRC treated with BVZ + chemotherapy**^11^.
**rs2305948**	***VEGFR2/KDR***	C/T	1.02 d	0.68–1.54	0.91	1.27 d	0.82–1.96	0.28	**Wild-type CC carriers have a higher tumour response in mCRC treated with BVZ + chemotherapy**^12^**, replication failed in**^9^.
						*Agreement for the TT genotypes:*			**T carriers associated with improved PFS in metastatic renal cell carcinoma, colorectal and breast cancers treated with chemotherapy + BVZ**^6^.
**rs11133360**	***VEGFR2/KDR***	C/T	0.88 r	0.56–1.40	0.60	**0.63 r**	**0.4–0.99**	**0.045**	
**rs9513070**	***VEGFR1/FLT1***	A/G	1.17 d	0.78–1.78	0.45	1.43 d	0.9–2.27	0.13	**AA carriers associated with longer PFS and OS in advanced CRC patients treated with BVZ + chemotherapy**^11^.
						*Inverse association:*			**G carriers have worse outcome in Renal Cell Carcinoma**^22^**, not replicated in mCRC**^9^**. Treatment in both cases: BVZ + chemotherapy**.
**rs7993418**	***VEGFR1/FLT1***	A/G	0.73 d	0.50–1.07	0.10	**0.7 d**	**0.47–1.05**	**0.086**	
						*Inverse association:*			**AA carriers associate with best and A carriers associated with better PFS and OS in pancreatic cancer**^22^ **and response rate in mCRC**^8^ **treated with BVZ + chemotherapy**.
**rs9582036**	***VEGFR1/FLT1***	A/C	0.8 d	0.56–1.16	0.24	**0.58 d**	**0.39–0.86**	**0.007**	
**rs2286455**	***CD133/PROM1***	C/T	**1.43 d**	**0.94–2.17**	**0.095**	**1.93 d**	**1.21–3.06**	**0.006**	**Correlated with PFS in combination with another SNP on the same gene**^27^ **in CRC treated with BVZ + chemotherapy**.
**rs833061**	***VEGFA***	T/C	1.06 d	0.70–1.06	0.78	1.03 d	0.67–1.58	0.89	**TT associated with superior overall response rate**^11^**, TT associated to shorter PFS**^9^ **in CRC treated with BVZ + chemotherapy**.

For *VEGFR2/KDR* rs11133360 C/T, TT carriers had improved OS (HR = 0.63, 95% CI: 0.40–0.99, *p-val* = 0.045) in APD, partially in line with our earlier study that any T at this locus was associated with improved PFS in mCRC, renal cell carcinoma and breast cancer patients treated with BVZ (^6,7,22^ and references therein). *VEGFR2/KDR* rs2305948 C/T mutant carriers, harbouring a Val273Ile substitution that reduces binding of VEGF-A to VEGFR2^23^, did not associate with outcome in APD. Gerger *et al*.^12^ reported that this polymorphism showed an association in Asian, but did not associate with outcome in Caucasian patients. *VEGFR1/FLT1* rs9582036 and rs7993418 and *VEGFA* rs833061 did not replicate in APD, while rs9513070 showed a same-sense association with OS as in^11^ without reaching significance, Table 2.

Interestingly, two inflammatory genes showed significant associations: *IL8* rs4073 T/A showed a significant association with PFS in APD: any A carriers for this SNP had worse PFS than TT carriers, (HR = 1.51, 95% CI: 1.03–2.22, *p-val* = 0.037), in line with previously reported results in CRC^10,11,24^ and ovarian cancer^25^. Furthermore, TT carriers for *TBK1* rs7486100 A/T, regulatory of tumor-associated macrophages, associated with worse PFS (HR = 1.94, 95% CI: 1.04–3.61, *p-val* = *0.037*) and OS (HR = 1.86, 95% CI: 0.94–3.68, *p-val* = *0.074*) in *KRAS* wt patients from APD, partially in line with results in^26^, where any T was associated with worse PFS in *KRAS* wt mCRC patients.

CC carriers of *CD133/PROM1* rs2286455 were associated to better OS (*p-val* = *0.006*) than any T carriers in APD, partially replicating the results from^27^, where *CD133* rs2286455 CC concomitant with *CD133* rs3130 CC where among the favourable allele combinations for PFS in mCRC patients treated with BVZ + chemotherapy.

Other polymorphisms which were associated with outcome in other BVZ studies, such as *VEGFA* rs699946 and rs699947^6^, *VEGFR2/KDR* rs12505758^6,9^, *CXCR2* rs2230054^12,25^, *MKNK1* rs8602^13^, *GC* rs4588^14^ and *CD133* rs3130^27^, were in regions not covered by our exome sequencing or not detected in our cohort.

### Identification of novel genetic variation candidates via machine learning

**Figure 1:**
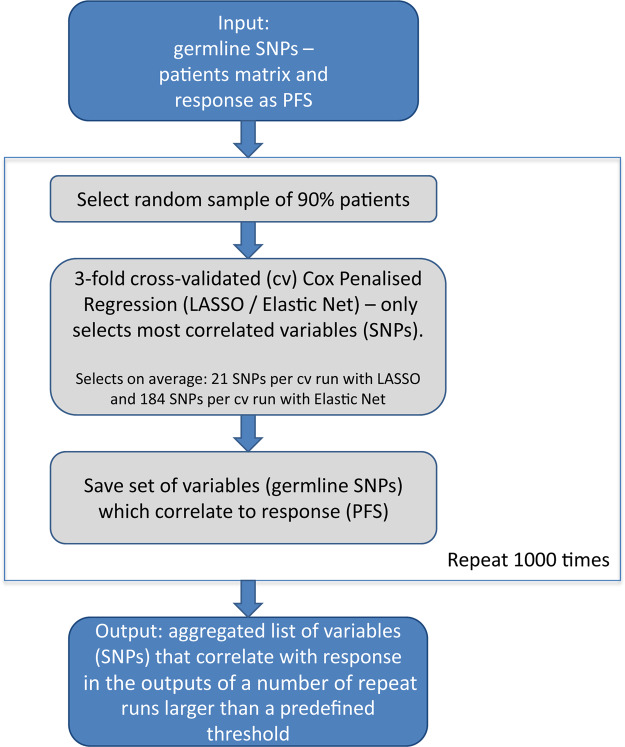
Work flow describing feature selection via repeated penalized regression.

Next, we extended our study in order to identify predictive SNPs across all exomes by machine learning, (Fig. 1). To curb the effect of multiple testing coupled with model overfitting, we used a repeated cross-validated approach, selecting candidate SNPs on the basis of them being predictive in multiple data subsets. Two approaches (LASSO and Elastic Net) of repeated three-fold cross-validated penalized regression were used for feature selection from the 74.648 germline SNPs detected with exome sequencing. Both algorithms were repeated 1000 times over randomly sampled patient sets encompassing 90% of the 180 BVZ-treated patients. The SNPs correlating with PFS in >25% LASSO- and >70% Elastic-Net-based repeat models were reported (see Methods). Altogether, 48 distinct SNPs were selected between the two repeated penalized regression approaches. Of these, one SNP (*CYP4F3* related) departed from Hardy-Weinberg equilibrium and thus was eliminated from the analysis. The remaining 47 SNPs and their characteristics (including multiple-testing correction of the log-rank test p-values) are given in the Suppl. Table S1. They comprised 6 SNP pairs in linkage disequilibrium (LD) and mapping to the same gene. The HRs, 90% CIs and Wald test p-values for these SNPs from Cox proportional hazards analysis adjusting for the relevant clinical covariates in the 180 mCRC APD patients are given in Suppl. Table S1 for all these SNPs, while Suppl. Fig. S1a illustrates comparatively, using bootstrapped Prediction Error Curves (PECs), that more variation in response (PFS) is accounted for by genetic variation (with models comprising from 2 to 30 of the SNPs) as opposed to the available clinical covariates and the null model. None of these SNPs had the same sense associations with outcome in the APD patients treated with chemotherapy-only, n = 16 (in Cox models involving each of the SNPs separately, adjusted for clinical covariates, data not shown). This is also illustrated in Suppl. Fig. S1b: for the chemotherapy-only setting, gradual incorporation of genetic variation to the model comprising only the clinical covariates equates with introducing additional noise, resulting in models with prediction errors exceeding those obtained using the baseline PFS probability function.

### Validation of novel genetic variation candidates identified by machine learning

Of the 47 SNPs selected by the ML approach, 34 were accessible for validation in two independent cohorts, MAVERICC and TRIBE. These cohorts have been selected based on the facts that, like APD, they both included a mix of *KRAS* mutant and wt patients, and like the majority of patients in APD (98%), were treated with first-line BVZ plus chemotherapy. All SNPs were in Hardy-Weinberg equilibrium in both validation cohorts, while genotype frequency was significantly different from the APD cohort in MAVERICC for 7 and in TRIBE for 9 SNPs, (Fisher’s Exact test *p-value* < 0.05, Suppl. Table S1). For each of the SNPs, we compared the associations between genotype and PFS in APD patients and the validation subgroups from MAVERICC and TRIBE cohorts, using Cox models adjusted for available clinical covariates. Of the 34 SNPs assessed, only 2 SNPs could be replicated in one of the validation cohorts: both *NLRP1* rs12150220 and *SRL* rs13334970 validated in MAVERICC (Suppl. Table S2), but not in TRIBE.

For rs12150220, missense at amino acid 155 of *NLRP1* (NLR Family Pyrin Domain Containing 1), carriers of 2 copies of the rare allele T had significantly better PFS than carriers of any A in both APD (HR = 0.52, 95% CI: 0.33–0.83, *p-val* = 0.006) and MAVERICC (HR = 0.42, 95% CI: 0.21–0.85, *p-val* = 0.017). Survival plots are shown in Suppl. Fig. S2. Carrying TT at this location reduced the hazard of relapse by ~50% in APD and ~60% in MAVERICC. In further exploratory analyses, we investigated the associations in the *KRAS* wt versus *KRAS* mutant patients in the three cohorts. We noticed that the association was conserved in the *KRAS* wt patients in both APD and MAVERICC (Suppl. Table S2) but lost in the MAVERICC *KRAS* mutant patients (Suppl. Table S3). In the TRIBE subgroup, decomposition according to *KRAS* status showed that rs12150220 TT carriers behaved similarly to any A carriers in the wt patients (Suppl. Table S2, Suppl. Fig. S2f) and had worse PFS than any A carriers in the *KRAS* mutant patients, Wald test *p-val* = 0.009 in Cox analysis adjusted for clinical covariates, (Suppl. Table S3). These results suggested that *NLRP1* rs12150220 TT carriers may have lower risk of relapse than any A carriers in wt *KRAS* mCRC patients.

An association between PFS and genotype was also shown for six consecutive downstream SNPs all mapping to *NLRP1*, all in linkage disequilibrium (LD), of which the immediate two downstream SNPs are also missense (rs2301582, rs11651270) and the next four SNPs are intron variants (rs12944976, rs12946467, rs59564976, rs56750129). All of these SNPs were highly correlated and co-inherited with rs12150220 [*min*(*r*)=0.76, *min*(*D’*) = 0.86, where *r* is the correlation coefficient and *D’* is the Scaled LD Estimate]. One of the missense variants, rs11651270 (Suppl. Table S1) was also selected by the ML approach, but did not validate in either of the two validation cohorts. All three non-synonymous mutations have been annotated with two algorithm tools used to predict the impact of non-synonymous SNPs on protein function: sorting intolerant from tolerant (SIFT)^28^ and polymorphism phenotyping (PolyPhen)^29^. rs12150220 is predicted deleterious by SIFT and benign by PolyPhen, while the other two mutations are predicted as tolerated by both algorithms. While in this context rs12150220 is likely the causal variant, the association of rs12150220 with PFS could well result from LD with another causal variant.

For rs13334970, mapping to *SRL* (Sarcalumenin), carriers of homozygous rare allele A at this location had shorter PFS than carriers of at least one allele G in both APD (HR = 2.3, 95% CI: 1.19–4.57, *p-val* = 0.014) and MAVERICC (HR = 2.5, 95% CI: 1.12–5.5, *p-val* = 0.025), see Suppl. Fig. S3a,b for survival plots. Being an AA carrier at this location increased the hazard of relapse by factors of 2.3 and 2.5 in APD and MAVERICC respectively. This association did not replicate in TRIBE, where AA carriers tended to behave inversely from APD and MAVERICC, (Suppl. Fig. S3c; Suppl. Table S2). Of note, genotype distributions for this SNP were statistically different in APD and TRIBE for the overall patients (Fisher’s Exact test, *p* = 0.003), but comparable for *KRAS* wt patients (*p* = 0.34). We thus assessed the same association in the *KRAS* wt patients. While the association maintained significance for APD and MAVERICC in the *KRAS* wt patients, any G and AA behaved similarly in TRIBE (Suppl. Table S2 and Suppl. Fig. S3f). In the *KRAS* mutant patients, the association lost its significance in both APD and MAVERICC and was statistically significantly inversed in TRIBE, (*p-val* = 0.019), Suppl. Table S3. These results taken together suggested that *SRL* rs13334970 AA carriers have increased risk of relapse compared to any G carriers in *KRAS* wt mCRC patients.

Sarcalumenin is a Ca^2+^-binding glycoprotein, which facilitates Ca^2+^ sequestration in the sarcoplasmic reticulum^30^. Together with rs13334970, another 11 SNPs were annotated to *SRL*, of which one 355 base pairs (bp) downstream (intronic variant rs13334805) was both co-inherited (*D’* = 0.99) and highly correlated (*r* = 0.99) with rs13334970. This suggested that rs13334805 may represent a substitution marker for rs13334970 and that the haplotype involving these 2 SNPs may be causative for the observed association.

For *STPB* rs229592 A/G, the negative association between *KRAS* wt rs229592 GG carriers and PFS (HR = 5.25, 95% CI: 1.94–14.23, *p-val* = 0.006) validated in the TRIBE cohort (HR = 10.3, 95% CI: 2.51–42, *p-val* = 0.005), however the GG numbers were low in both APD (seven) and TRIBE (three) *KRAS* wt cohorts, Suppl. Table S4. This locus encodes a member of the spectrin family, which plays a role in cell membrane organization and stability.

### Combination of *NLRP1* rs12150220 and *SRL* rs13334970

**Figure 2:**
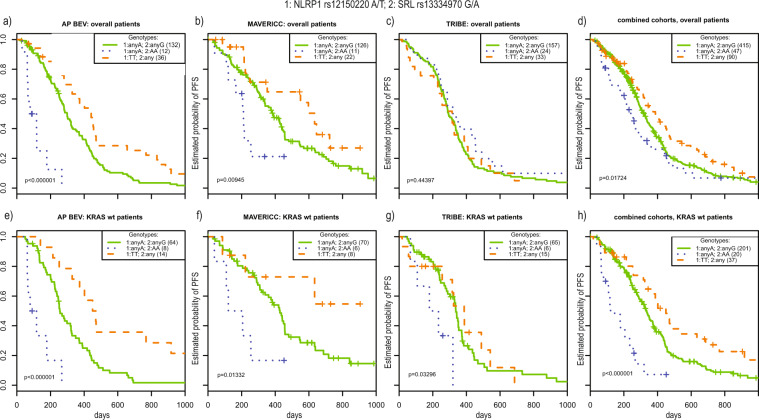
Kaplan Meier plots for the combined genotypes of SNPs rs12150220 (*NLRP1*) and rs13334970 (*SRL*). X-axis: days from randomization, Y-axis: estimated probability of progression free survival (PFS). Log-rank test p-values are given for each KM plot. Individuals carrying at least one major allele for each of these two SNPs constituted the largest group, which was used as baseline in Cox proportional hazards modelling (continuous line). Individuals homozygous on the rare allele A for *SRL* rs13334970 and carrying at least one major allele A for *NLRP1* rs12150220 - dotted line. Individuals homozygous on the rare allele T for *NLRP1* rs12150220 – interrupted line. (**a**) APD – overall patients. (**b**) MAVERICC – overall patients. The combination validates in this subgroup. (**c**) TRIBE – overall patients. No validation in this subgroup. (**d)** The 3 cohorts combined – overall patients. (**e**) APD *KRAS* wt. (**f**) MAVERICC *KRAS* wt. (**g**) TRIBE *KRAS* wt. (**h**) The 3 cohorts combined – *KRAS* wt patients. There was validation for negative association between the individuals homozygous on the rare allele A for *SRL* rs13334970 and at least one major allele A for *NLRP1* rs12150220 and PFS in both MAVERICC and TRIBE *KRAS* wt patients, with the association retaining significance when combining the 3 cohorts.

We finally looked at the combination of *NLRP1* rs12150220 and *SRL* rs13334970 in the overall patient cohort and, based on the above data, specifically in the *KRAS* wt patients. Figure 2 shows the KM plots of three groups of genotypes based on combinations of rs12150220 and rs13334970 genotypes. For the overall patients in APD, carriers of TT for *NLRP1* rs12150220 did significantly better (HR = 0.52, 95% CI: 0.33–0.83, coefficient-wise *p* = *0.006*) regardless of their *SRL* rs13334970 allele, while *NLRP1* rs12150220 any A and *SRL* rs13334970 AA carriers had significantly worse PFS (HR = 8.3, 95% CI: 3.3–21, *p* = 7 × 10^−6^), with all other combinations as baseline (concomitant *NLRP1* rs12150220 any A and *SRL* rs13334970 any G). Suppl. Fig. S1a shows how much variation in response (PFS) is accounted for by these 2 variants (model.2.SNPs) as opposed to the available clinical covariates in the bootstrapped APD overall cohort. These associations validated for the overall patients in MAVERICC (better outcome for *NLRP1* rs12150220 TT carriers, (HR = 0.45, 95% CI: 0.22–0.91, *p* = 0.026) and worse outcome for *NLRP1* rs12150220 any A and *SRL* rs13334970 AA carriers, (HR = 2.2, 95% CI: 1–5, *p* = 0.05), but not for the overall patients in TRIBE, (Fig. 2b,c; Suppl. Table S5). In the *KRAS* wt patients, the negative association between PFS and concomitant carriers of *NLRP1* rs12150220 any A and *SRL* rs13334970 AA was maintained in APD (HR = 4.4, 95% CI: 1.2–16.1, *p* = 0.02, overall Wald test *p-value* = 0.005). As expected from the above exploratory analysis, this association validated in both MAVERICC *KRAS* wt (HR = 3, 95% CI: 1.2–8, *p* = 0.025) and TRIBE *KRAS* wt (HR = 7.8, 95% CI: 2.5–24.4, *p* = 0.00045), Fig. 2f,g. Given the low frequency of patients carrying the *NLRP1* rs12150220 any A/*SRL* rs13334970 AA genotype, we also conducted an analysis of the combined datasets (APD, MAVERICC and TRIBE), Fig. 2d,h. The association between this genotype and outcome was also significant (HR = 3.6, 95% CI: 2.1–6.1, *p* < 10^–4^), Suppl. Table S5. In the combined analysis for the *KRAS* wt patients, the *NLRP1* rs12150220 any A / *SRL* rs13334970 AA carriers showed a remarkably shorter PFS than the other two genotype combinations with better outcomes (4 vs 11 and 15 months median PFS, *p* < 10^−4^) and also shorter OS (12 vs 27 months median OS, *p* = 0.04).

## Discussion

In this study we have investigated previously discovered SNPs associated with BVZ-responsiveness, and used a machine-learning approach to select new candidate germline SNPs. We have used the germline SNP data of 180 mCRC patients treated with BVZ plus chemotherapy, obtained with exome sequencing in the APD project, to replicate SNPs that have been proposed as candidate biomarkers for response to BVZ plus chemotherapy in other studies. Of 11 variants assessed, we could replicate statistically significant same-sense association between the SNP and treatment response (PFS or OS) for *VEGFR2/KDR* rs11133360 and *CD133/PROM1* rs2286455, and for two SNPs in pro-inflammatory genes: *IL8* (rs4073; all patients) and *TBK1* (rs7486100; *KRAS* wt patients). Of note, *IL8* rs4073 represents one of the few SNPs that have been replicated in more than one metastatic colon cancer study. *IL8*, besides showing angiogenic activity, is a major mediator of the inflammatory response. *IL8* rs4073 A carriers, which associate with worse outcomes, exhibited significantly higher baseline *IL8* serum levels in a cohort of *KRAS* mutant mCRC patients^24^. TBK1 (NF-κB-Activating Kinase) plays an essential role in regulating inflammatory responses, as it associates with TRAF3 and TANK to activate NF-κB and phosphorylates interferon regulatory factors (IRFs). This activity allows nuclear translocation of the IRFs leading to transcriptional activation of pro-inflammatory and antiviral genes including *IFNA* and *IFNB*. Hence differences in inflammatory signaling in the tumour microenvironment appears to play a prominent role in responses to BVZ plus chemotherapy, as recently also identified by next generation sequencing and immunoprofiling analyses of metastatic CRC tumors^31^.

Of the 34 novel SNPs selected by ML from the APD cohort which were assessed for validation in two independent cohorts, two SNPs, *NLRP1* rs12150220 and *SRL* rs13334970, were validated in the MAVERICC cohort for the overall patient cohort, one of which also encoding for a pro-inflammatory gene (*NLRP1*). These associations were conserved in the *KRAS* wt patients in both APD and MAVERICC, but partially lost (*NLRP1* rs12150220) in the MAVERICC *KRAS* mutant patients. Interestingly, when combining the effects of these two SNPs, we found a negative association between PFS and *NLRP1* rs12150220 any A and *SRL* rs13334970 AA concomitant bearers that validated in both MAVERICC and TRIBE cohorts for *KRAS* wt patients, and in the combined cohorts. While the polymorphism *SRL* rs13334970 has a statistically different distribution of genotypes in TRIBE compared to APD for the overall patients (Fisher’s exact test *p-value* = 0.003), the two cohorts are actually comparable for the *KRAS* wt patients only, for this same polymorphism, (*p-value* = 0.34). Therefore, our finding that the combination of *NLRP1* rs12150220 and *SRL* rs13334970 only validated in the TRIBE *KRAS* wt patients (Suppl. Table S5) may be related to this specific genotype distribution at *SRL* rs13334970 in TRIBE. Moreover, although *KRAS* status had no impact on outcome in the APD cohort (reference: *KRAS* wt; PFS: HR = 1.06, 95% CI: 0.72–1.58, *p-val* = 0.75; OS: HR = 0.74, 95% CI: 0.48–1.14, *p-val* = 0.17), patients with *KRAS* mutant tumors had less benefit from BVZ than *KRAS* wt patients in TRIBE^32^, which may also explain our findings. Although *NLRP1* rs12150220 and *SRL* rs13334970 did not associate with survival in the chemotherapy-only-treated mCRC patients of the APD cohort, the small number of patients (n = 16) warrants further validation in a larger chemotherapy-only treated group.

rs12150220 on *NLRP1* is a missense variant at amino acid 155 (His to Leu) of NLRP1 (NLR Family Pyrin Domain Containing 1). This protein mediates inflammasome activity in response to damage-associated signals through activation of Caspase-1, playing a key role in innate immunity and inflammation. Following activation, NLRP1 can directly interact with Caspase-1 to form a functional inflammasome, a molecular complex that participates at maturation and release of cytokines including interleukin-1β^33^. NLRP1 also collaborates with Apoptotic Peptidase Activating Factor 1 (APAF1) to induce caspase activation and apoptosis in intact cells^34^, suggesting interaction between host defence and apoptosis machinery, presumably allowing for coordination of cell death and host defence^33^. Interestingly, among the SNPs that were outlined as strongly associated with PFS by our ML approach, rs7104785 mapped to *PIDD* (p53-induced protein with a death domain). PIDD activates Caspases-2, -3 and -7 and is structurally similar to *NLRP1* C terminus in that an LRR (leucine-rich repeats) and a CARD (caspase activation and recruitment domain), flank the site of proteolysis^35^. However, the association between this variant and PFS (data not shown) could not be replicated in the two validation cohorts.

The second new germline SNP emerging from this study, rs13334970, maps to *SRL* (Sarcalumenin), a Ca^2+^-binding glycoprotein, which facilitates Ca^2+^ sequestration in the sarcoplasmic reticulum^30^, a subcompartment of Endoplasmic Reticulum (ER)^36^. ER is the major site of calcium storage and protein folding. Misfolded protein aggregates trigger ER stress, known to produce pro-inflammatory signals, and to activate stress signalling and apoptosis^37^. Interestingly, senescent *SRL* knock-out mice exhibited increased ER stress^38^ suggesting *SRL* has a role in ER stress signalling. *SRL* rs13334970 is coinherited and highly correlated with another intronic variant *SRL* rs13334805. Intronic variants can affect the phenotype in many ways, for example by disrupting intron transcription regulatory motifs, inclusion of pseudo-exons or else competition with natural splice sites, possibly leading to altered SRL protein levels or function^39^.

A limitation to our findings is that, apart from *NLRP1* rs12150220 and *SRL* rs13334970, the associations for the other 32 SNPs, that were tested in the two validation cohorts, could not be replicated. However, one key difference that may preclude a direct comparison of the cohorts is the chemotherapy backbone. While the treatment was homogeneous across the present TRIBE and MAVERICC subgroups (FOLFIRI + BVZ), most patients in APD had an oxaliplatin-based chemotherapy backbone. Differences also exist within TRIBE and MAVERICC regarding tumor location (in MAVERICC more patients had their primary tumour on the right side) and number of metastases (MAVERICC had less patients with 2 or more metastases). Furthermore in MAVERICC, most patients did not have their primary tumour resected, Table 1. This clinical information was not available for APD. Both APD and TRIBE are European cohorts while the MAVERICC cohort is American (including Hispanic and Asian Americans). BVZ may have varying efficacy among different ethnic groups^40^, and allelic frequencies of polymorphisms may differ between ethnic groups and influence clinical effects^12^. Despite the differences across the three subgroups, it is noteworthy that PFS associations for the *SR*L and *NRLP1* combination were consistent across the *KRAS* wt patients in all three cohorts. We have limited our feature selection approach to a manageable number of plausibly associated SNPs, of but it is not excluded that other clinically relevant variants could be detected and validated using less stringent selection criteria.

Our study is limited by the fact that only a relative small cohort of chemotherapy-only treated patients were included in the Angiopredict study, which prevents from concluding on the predictive vs prognosic value of the signature. Through validation of previously identified SNPs, and identification of novel SNPs on *NLRP1* rs12150220 and *SRL* rs13334970, our findings nevertheless highlight the importance of germline variations in genes encoding for pro-inflammatory proteins and ER proteins in responses of mCRC to BVZ + chemotherapy. Further validation of these SNPs, including using a larger chemotherapy-only group, may be warranted to convincingly demonstrate the predictive vs. prognostic potential of the identified SNP combination. Specifically, this study indicates that *KRAS* wt patients bearing the less favourable combination for the two SNPs discussed (AA for *SRL* rs13334970 and any A for *NLRP1* rs12150220) should not be considered for first-line treatment with chemotherapy plus BVZ, they should rather receive chemotherapy plus an anti-EGFR for example (subject to further research), while *KRAS* wt patients carrying TT for the inflammasome-related gene *NLRP1* rs12150220 could benefit from first-line chemotherapy plus BVZ. Our study also highlights the interest of ML approaches in identifying novel genomic biomarkers of drug responsiveness and the importance of clinical validation of genetic variation findings.

## Materials and Methods

### ANGIOPREDICT (APD) cohort

Patients with advanced (locally inoperable or metastatic) CRC receiving chemotherapy alone or in combination with BVZ (first, second and higher lines) between July 2004 and April 2012 were included in this analysis. The data were retrospectively collected by reviewing patient records within four different European cohorts (Table 1), including three single center cohorts^17^ and a multi-center subgroup of patients selected from the CAIRO2 trial^41^. A total of 180 patients were treated with BVZ + chemotherapy and 16 patients - with chemotherapy-only.

Informed consent was obtained from each patient and the study was carried out in accordance with the relevant guidelines and regulations effective for the 3 participating study centers. Specifically, ethical review and approval of health-related research studies which are not clinical trials of medicinal products for human use as defined in SI 190/2004 was undertaken by the Research Ethics Committee of Beaumont Hospital, RCSI Hospital Group in Ireland. The study was approved by the Medical Ethics Commission of the Faculty of Medicine in Mannheim, Heidelberg University in Germany. The collection, storage, and use of archival tissue and patient data were performed in compliance with the Code for Proper Secondary Use of Human Tissue in The Netherlands (2011), developed by the standing committee of the FEDERA (FMWV Foundation, Chamber of Commerce, Rotterdam, 41055219, http://www.fmwv.nl and www.federa.org) and the Commissie Regelgeving in Onderzoek (COREON), in close cooperation with BBMRI-NL and patient organisations.

### Validation Cohorts

Germline mutations have been determined using the OncoArray, a customized array manufactured by Illumina (San Diego, CA, USA) including approximately 530 K SNP markers^42^ in two independent validation subgroups of patients, Table 1. The first validation cohort consisted of 163 mCRC patients treated with first-line FOLFIRI + BVZ from the randomized phase 2 study MAVERICC^43,44^. This study included both *KRAS* mutant (codons 12 and 13) and wt patients. The second validation cohort consisted of 215 mCRC patients treated with first-line FOLFIRI + BVZ from the open-label, phase 3, randomized TRIBE study^32^, comprising both *KRAS* mutant (codons 12, 13 and 61) and wt patients. In both validation cohorts, genomic DNA was extracted from blood samples using the QIAmp DNA easy kit (Qiagen, Valencia) and then genotyped through the OncoArray.

### Exome sequencing

Germline SNPs in APD were analysed by exome-sequencing of normal tissue from 196 patients. We designed a custom exome capture kit, which in addition to the regular exome also captured promoter regions and 5′ or 3′UTRs from a number of key angiogenic factors, covering ~14 K additional SNPs. We performed whole-exome sequencing using the Nimblegen SeqCapV3 exome capture kit (Roche Sequencing Inc.) applied to the Illumina HiSeq2000 sequencing platform (in a 2 × 100 bp paired-end run).

### Germline SNP calling—bioinformatics

In APD, on average 40 ± 20 million reads per sample were generated. The raw sequencing reads were mapped to the human reference genome (NCBI37/hg19) using Burrows-Wheeler Aligner (BWA v0.5.8a)^45^. Picard (v1.43) was used to remove PCR duplicates, resulting in an average coverage per sample of 37 ± 25X. The Genome Analysis Tool Kit (GATK v3.3)^46^ was used for local realignment around insertions and deletions and base recalibration. After this quality assessment, mapped germ-line sequencing data were available for 196 patients and an initial number of 441.440 germline SNPs were called. Common germline SNPs were selected using the following criteria: (1) The SNP had to be present in at least 10% and less than 100% of all the patients. (2) The SNP had to be present in at least 5% of the European individuals of the 1000 genomes project. (3) The position of the SNP had to be covered in at least 90% of the patients. 4) The SNP was annotated with a unique gene using Variant Effect Predictor (VEP) 8.1^47^. 74.648 final SNPs responding to the aforementioned criteria remained in the analysis. In the validation cohorts, SNPs that were not covered in at least 80% samples were excluded from analysis.

### Feature selection via repeated penalized regression

To select candidate SNPs from 74.648 featuring SNPs, a machine learning (ML) approach based on cross-validated Cox penalized regression was applied, with PFS as outcome variable. Two penalized regression algorithms were used: LASSO^48,49^ and Elastic Net^50^, which combines the L2 regularization (used in ridge regression) and L1 regularization (used in LASSO). Both Lasso and Elastic Net are efficient methods to perform variable selection in high-dimensional data settings with relatively few observations^50^, as the case is here. The Elastic Net algorithm depends on a parameter α comprised between 0 and 1. The number of selected variables (SNPs here) increases when α is close 0, decreases as α approaches 1 and LASSO is performed when α = 1. The value of α was set to 0.5 for this study.

Three-fold cross-validated (cv) Cox LASSO and Elastic Net regression respectively were repeated on 1000 random subsets of 90% of the samples, using the Bioconductor R package *glmnet*. Each three-fold cv Cox regression repeat selects a list of SNPs that result in a minimum mean cross-validated error. The cv method of penalized regression performed repetitively on smaller fractions of the dataset selects the features that are correlated to the outcome (PFS) without over-fitting to our specific sample set. For LASSO, where fewer variables per repeat are selected (on average 21 germline SNPs), variables selected in more than 250 of 1000 cv repeat models were reported at the end of the process. Elastic Net tends to select considerably more variables: in our case (α = 0.5), on average 184 variables were selected per cv repeat. Therefore, to limit the selected variables to the most correlated over the entire dataset, only variables selected in 700 or more cv repeat models were aggregated and reported at the end of the process, (Fig. 1). These thresholds were chosen to support a reasonable trade-off between number of SNPs selected and their likelihood of real association to PFS.

### Statistical analysis

PFS was the primary end-point of the analysis and was defined as the time from start of treatment (chemotherapy + BVZ or chemotherapy-only) to relapse (progressive disease) or death from any cause, whichever occurred first. Patients stopping BVZ therapy due to other reasons than progression or death were censored at date of treatment stop. OS was defined as time from start of BVZ to death from any cause.

Cox proportional hazards regression analysis (Cox models) were used to correlate each SNP selected by ML with PFS and estimate genotype-specific hazard ratios (HR) and 95% confidence intervals (CI), adjusting for the available covariates [sex, age, grade of differentiation, chemotherapy line (1^st^ vs grouped 2^nd^ and higher), chemotherapy backbone administered together with BVZ (backbone containing a fluoropyrimidine vs non-standard backbones not including a fluoropyrimidine^17^ and *BRAF* codon 600 V/E status (determined by exome sequencing in tumor and normal paired samples)] for the retrospective APD cohort. For the patients in the validation cohort MAVERICC, Cox models were adjusted for age, ECOG performance status, number of metastases, and resection of the primary tumor. For the patients in the validation cohort TRIBE, Cox models were adjusted for age, sex, ECOG performance status, primary tumour site, resection of the primary tumour, liver limited disease, adjuvant chemotherapy, *BRAF* status, and *RAS* status. For each of the candidate SNPs, HR and CI were generated using codominant, dominant and recessive models for the discovery and validation cohorts, respectively. The Wald p*-*values (*p-val*) presented for Cox models correspond to the significance of the Wald test across the three genotype groups (common allele homozygote, heterozygote, rare allele homozygote) or the two genotype groups for the dominant and recessive models. For each HR specifically, the order of significance of the HR being different from 1 is also given, representing the probability *p* that the estimated Cox model coefficient could be zero. The order of magnitude of *p* is given by asterisks in the tables. The threshold for all p-values was set to 0.05.

Kaplan-Meier (KM) plots and log-rank tests were also used for each individual SNP selected with ML. The log-rank test p-values were corrected for multiple-testing, using a permutation method. The multiple-testing corrected p-value is given by the frequency of obtaining a p-value of this order or lower randomly (using a permutated genotypes variable), with 10,000 permutations being employed. The performance of distinct multivariate Cox models was evaluated based on computing the Brier scores. The Brier score is a weighted average of the squared distances between the observed survival status and the predicted survival probability of a model. Prediction Error Curves (PECs) are finally obtained when the Brier score is followed over time.

In all analyses, the SNPs required a *p-value* > 1 × 10^−4^ when departing from the Hardy-Weinberg equilibrium. All statistical analyses were undertaken using R, using libraries *survival* (KMs, Cox models) and *pec* (Prediction Error Curves).

### Statement of translational relevance

Investigating germline single-nucleotide polymorphisms (SNPs) in the Angiopredict [APD] cohort, our study highlights the importance of inflammatory signaling in the tumor microenvironment in the clinical response of metastatic colorectal cancer patients to the angiogenesis inhibitor bevacizumab. From a methodological perspective, our study highlights the significance and demonstrates the limitations of machine learning approaches for the identification of novel genomic biomarkers of therapy responsiveness. From a clinical perspective, our study identifies a novel combination of two germline SNPs that are associated with unfavorable responses to bevacizumab plus chemotherapy, a finding that was validated in two independent clinical cohorts for *KRAS* wild-type patients. Our data suggest that bevacizumab should not be considered as first-line treatment in patients carrying this germline SNP combination.

## Supplementary information

Supplementary Information .

Supplementary Table S1.

## Data Availability

The exome sequencing data are deposited at the EMBL-EBI under accession code EGAS00001002617 and are available under restricted access.
