# Supplementary material for: Combination of variations in inflammation- and endoplasmic reticulum-associated genes as putative biomarker for bevacizumab response in KRAS wild-type colorectal cancer
Source: Sci Rep. 2020 Jun 17;10:9778. doi: 10.1038/s41598-020-65869-2 (PMC7299973; doi:10.1038/s41598-020-65869-2)
Supplement: Supplementary file 1 — Supplementary Information . [file 41598_2020_65869_MOESM1_ESM.pdf]

Combination of variations in inflammation- and endoplasmic reticulum-associated genes as putative biomarker for bevacizumab response in *KRAS* wild-type colorectal cancer - Supplementary Figures and Tables

Ana Barat[1], Dominiek Smeets[2], Bruce Moran[3], Wu Zhang[4], Shu Cao[4], Sudipto Das[5], Rut Klinger[6], Johannes Betge[7,8], Verena Murphy[9], Orna Bacon[1], Elaine W. Kay[10], Nicole C. T. Van Grieken[11], Henk M.W. Verheul[12], Timo Gaiser[13], Nadine Schulte[7], Matthias P. Ebert[7], Bozena Fender[14], Bryan T. Hennessy[15], Deborah A. McNamara[16], Darran O'Connor[5], William M. Gallagher[3], Chiara Cremolini[17], Fotios Loupakis[18], Aparna Parikh[19], Christoph Mancao[20], Bauke Ylstra[11], Diether Lambrechts[2], Heinz-Josef Lenz[4], Annette T. Byrne[1], and Jochen H.M. Prehn[1]

[1]Centre for Systems Medicine and Department of Physiology & Medical Physics, Royal College of Surgeons in Ireland, Dublin, Ireland

[2]VIB Vesalius Research Center, KU Leuven, Leuven, Belgium

[3]UCD Conway Institute, University College Dublin, Dublin, Ireland

[4]USC Norris Comprehensive Cancer Center, Los Angeles, USA

[5]Molecular and Cellular Therapeutics, Royal College of Surgeons in Ireland, Dublin, Ireland

[6]UCD, School of Biomolecular and Biomedical Science, Dublin, Ireland

[7]Department of Medicine II, University Hospital Mannheim, Medical Faculty Mannheim, Heidelberg University, Mannheim, Germany

[8]German Cancer Research Center (DKFZ), Division Signaling and Functional Genomics, Heidelberg, Germany

[9]Cancer Trials Ireland, Dublin, Ireland.

[10]Department of Pathology, Beaumont Hospital, Dublin, Ireland

[11]Department of Pathology, VU University Medical Center, Amsterdam, The Netherlands

[12]Department of Medical Oncology, VU University Medical Center, Amsterdam, The Netherlands

[13]Institute of Pathology, University Hospital Mannheim, Medical Faculty Mannheim, Heidelberg University, Mannheim, Germany

[14]OncoMark Ltd., NovaUCD, Belfield Innovation Park, Dublin, Ireland

[15]Department of Medical Oncology, Beaumont Hospital, Dublin, Ireland

[16]Department of Surgery, Beaumont Hospital, Dublin, Ireland

[17]Unit of Medical Oncology 2, Department of Translational Research and New Technologies in Medicine and Surgery, Azienda Ospedaliera Universitaria Pisana, Pisa, Italy.

[18]Oncology Unit, Istituto Oncologico Veneto, IOV-IRCCS, Padua, Italy.

[19]Division of Hematology and Oncology, Massachusetts General Hospital, Boston, USA.

[20]Oncology Biomarker Development, Genentech Inc., USA.

Correspondence to anabarat27@gmail.com and jprehn@rcsi.ie

Supplementary Figure S1. Prediction Error Curves (PECs) computed up to two years for nested models featuring 30, 25, 15, 10, 5 and 2 of the SNPs selected using penalized regression (see Methods). Models were assessed in a) 180 APD chemotherapy + BVZ patients, using bootstrap cross-validation and b) 16 APD chemotherapy-only patients.

Starting with a model involving all 30 SNPs, models comprising less SNPs were subsequently obtained by discarding the desired number of least significant SNPs, with all coefficients of models 15, 10 and 5 being described by p-values $p<0.05$. The SNPs involved in models 30, 15, 10 and 5 are available in Suppl. Table S1. The SNPs in the model with 2 SNPs are *NLRP1* rs12150220 and *SRL* rs13334970. The 'clinical covariates' model involves only the relevant clinical covariates. The Null Model corresponds to the baseline estimated PFS and includes no variables. For the APD patients treated with BVZ the model involving 25 SNPs had the best prediction errors for PFS around one year from study entry and adding in more variables did not improve the prediction error. The SNPs selected for the BVZ setting are not predictive in the chemotherapy-only setting, with the large prediction errors in panel b) indicating that the SNPs only introduce noise on the top of the baseline data.

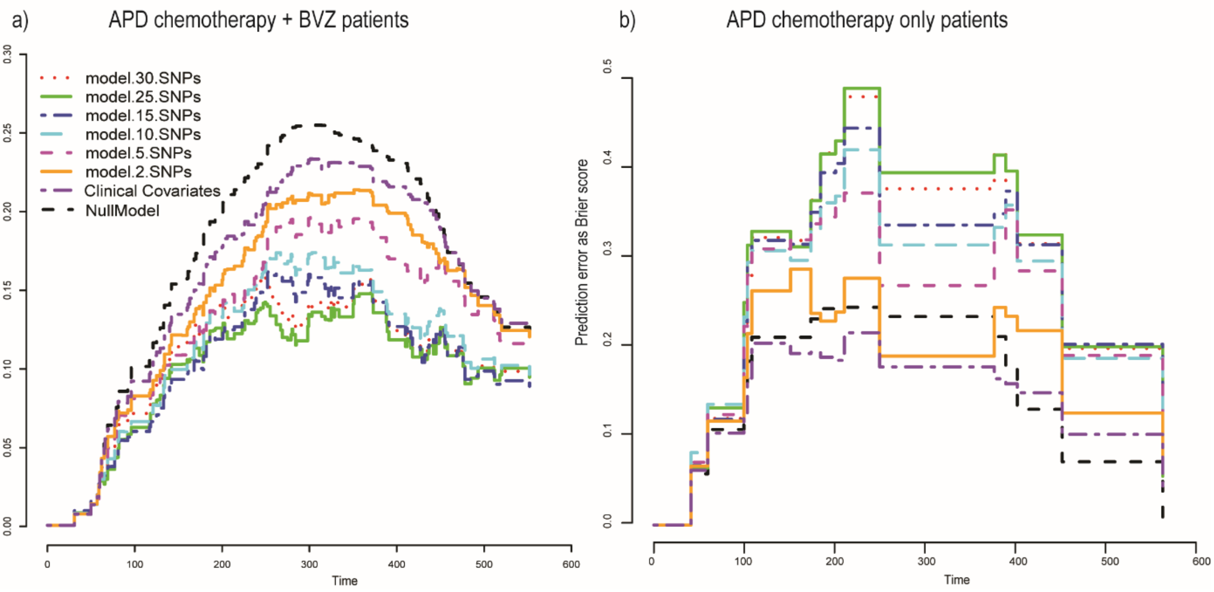

Supplementary Figure S2. Kaplan Meier plots for the genotypes of *NLRP1* rs12150220, a missense variant on the NLR Family Pyrin Domain Containing 1 gene, in a) ANGIOPREDICT patients treated with BVZ + chemotherapy; b) patients from the MAVERICC cohort, treated with combination chemotherapy (FOLFIRI) and BVZ. c) patients from the TRIBE-A cohort, treated with combination chemotherapy (FOLFIRI) and BVZ. d – f) *KRAS* wt patients in the same respective cohorts, any A versus TT. g – i) *KRAS* mutant patients in the same respective cohorts, any A versus TT. X-axis: days from randomization, Y-axis: estimated probability of progression free survival (PFS). Log-rank test p-values *p* are given for each KM plot.

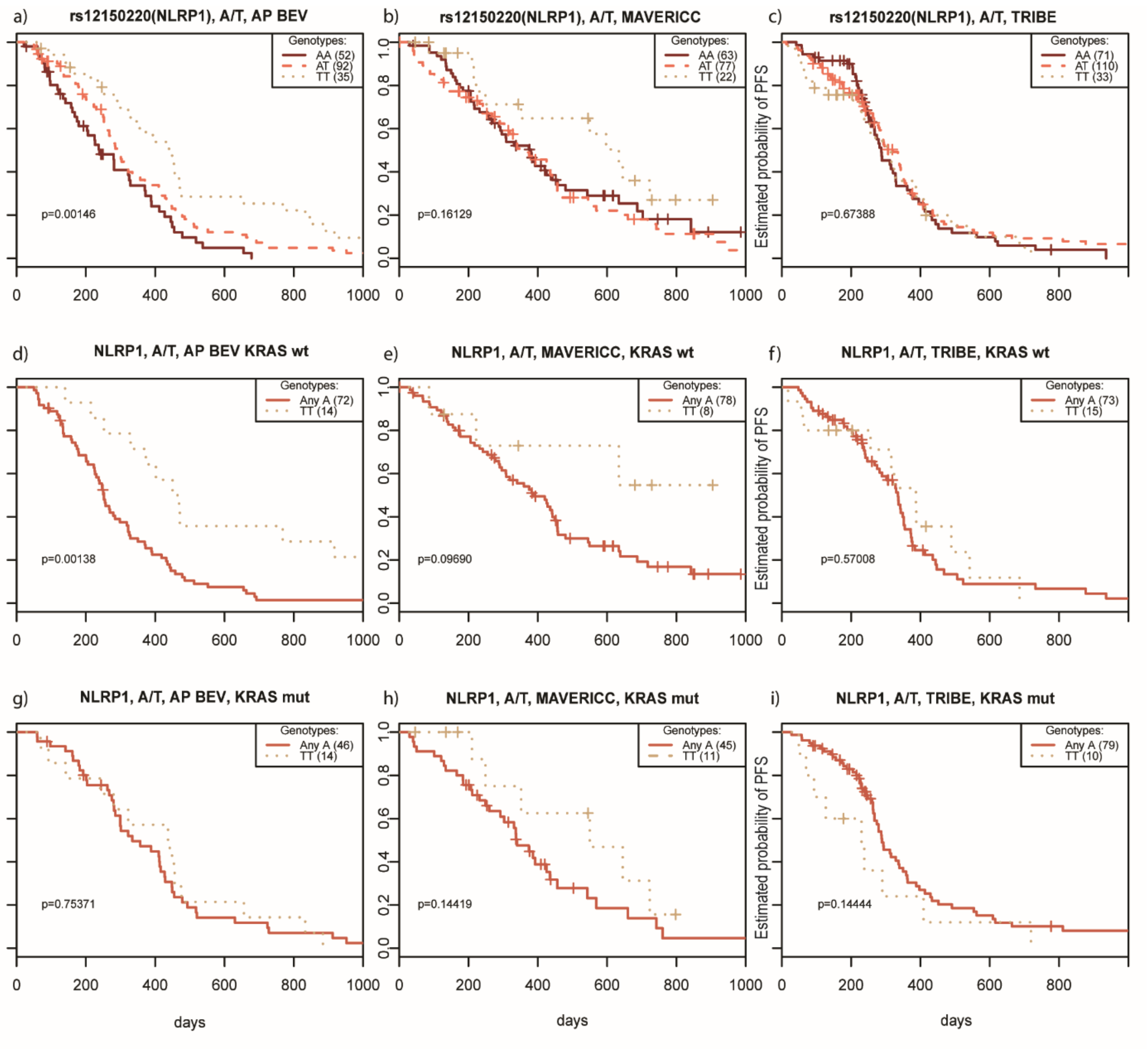

Supplementary Figure S3. Kaplan Meier plots for the genotypes of *SRL* rs13334970 mapping to an intronic region on the Sarcalumenin (*SRL*) gene in a) ANGIOPREDICT patients treated with BVZ + chemotherapy; b) patients from the MAVERICC cohort, treated with BVZ + chemotherapy; c) patients from the TRIBE cohort, treated with BVZ + chemotherapy. d – f) *KRAS* wt patients in the same respective cohorts, any G versus AA. KM plots not shown for *KRAS* mutants as low numbers for AA carriers in both Angiopredict and MAVERICC. X-axis: days from randomization, Y-axis: estimated probability of progression free survival (PFS). Log-rank test p-values are *p* given for each KM plot.

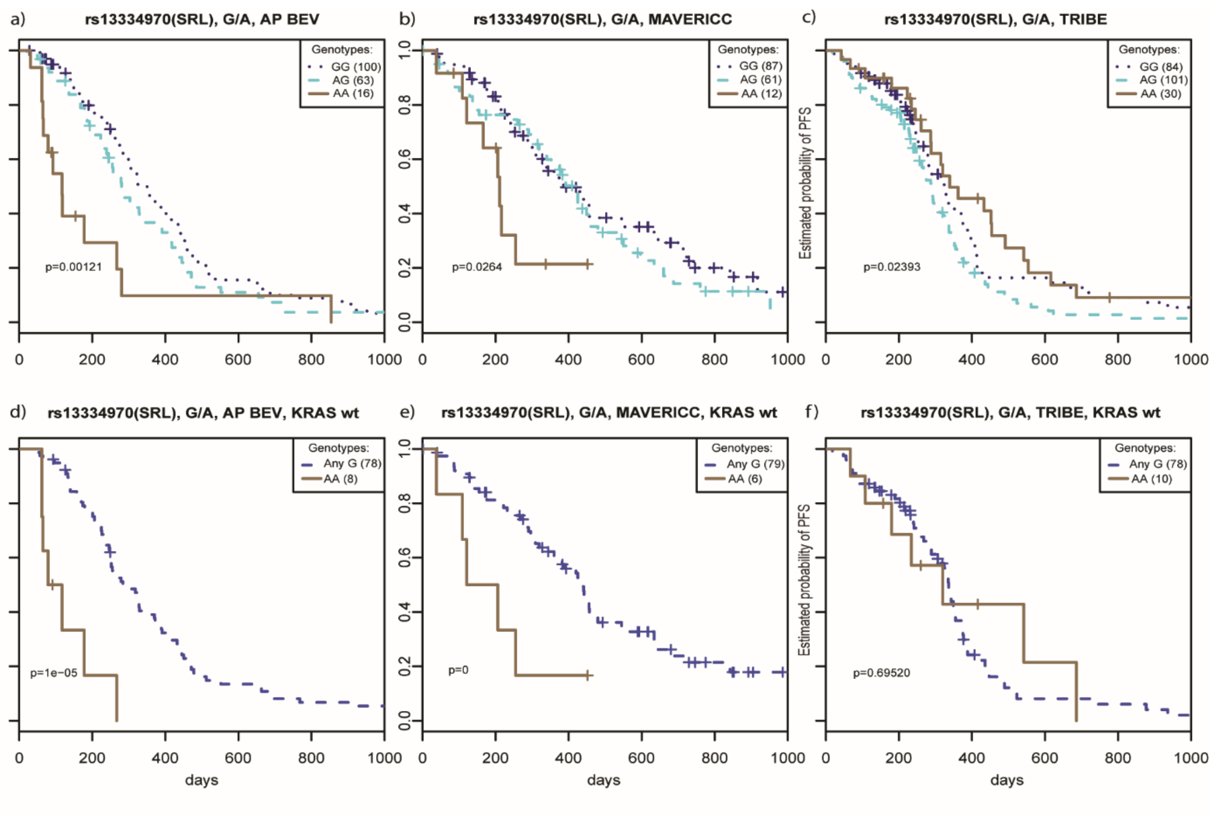

Supplementary Table S1. 47 SNPs that have been selected to be associated with progression-free survival, using repeated penalized regression as outlined in Methods, in metastatic or locally advanced colorectal cancer patients after treatment with BVZ combination therapy (ANGIOPREDICT retrospective cohort). Hazard ratios given on the table were computed using Cox proportional hazards analysis **for all patients treated with BVZ**, adjusting for age, gender, grade of differentiation, chemotherapy line, chemotherapy type and status of somatic BRAF codon 600 V/E. HR_{het} and HR headings designate the hazard ratios for the heterozygous and homozygous on the minor allele genotypes respectively, compared to baseline (homozygous on the major allele genotypes), when a codominant model was most significant. When a dominant (d) or a recessive (r) model were most significant, the respective hazard ratios are given under the HR heading. The HRs for each included SNP represent the exponentiated coefficients computed in the respective multivariate Cox model and are interpretable as multiplicative effects on the hazard: the % by which the risk of relapse is increased with change to one alternative genotype (in keeping all other considered features to baseline). Thus, for example, a patient carrying at least one alternative allele C for rs7104785 on PIDD increases the risk of relapse by ~60% considering a dominant model for this SNP. The order of magnitude of the probability *p* that the model coefficient for the indicated genotype could be zero (the hazard ratio could be equal to one) is indicated with **.**(dot) $0.05<p<0.1$; * $0.01 \le p \le 0.05$; ** *p* of order 0.001; *** *p* of order 0.0001 and less. If no order of magnitude for *p* is given then $p \geq 0.1$ and is not statistically significant.

Provided as Excel File.

Supplementary Table S2. *NLRP1* rs12150220 and *SRL* rs13334970 in APD, MAVERICC and TRIBE cohorts, overall and *KRAS* wt respectively. The associations found in APD validate in MAVERICC. In TRIBE *KRAS* wt, the same sense HR is found for *SRL* rs13334970 AA carriers as in the other two cohorts, without reaching statistical significance. HRs, the respective 95% CI and the across-genotype Wald test p-values are presented under codominant, dominant and recessive models, using multivariable Cox proportional hazard regression models adjusted for available (see Methods) covariates. For each HR specifically, the order of significance of the HR being different from 1 is given by stars. The stars give the order of magnitude of the probability *p* that the model coefficient for the indicated genotype could be zero (the hazard ratio could be 1), with * *p* of order 0.01, ** *p* of order 0.001, *** *p* of order 0.0001.

		ANGIOPREDICT overall<br>*Progression-free Survival*			MAVERICC overall<br>*Progression-free Survival*			TRIBE overall<br>*Progression-free Survival*		
SNPs	*Genotype*	*n*	*Adjusted HR (95%CI),p (stars).*	*Wald test p-value*	*n*	*Adjusted HR (95%CI)*	*Wald test p-val*	*n*	*Adjusted HR (95%CI)*	*Wald test p-value*
**rs12150220 (*NLRP1*)** codominant	**A/A**	52	Reference	**0.011**	63	Reference	**0.042**	71	Reference	0.47
	**A/T**	92	0.78(0.51,1.19)		77	1.18(0.76,1.82)		110	0.93(0.62,1.41)	
	**T/T**	36	0.43(0.25,0.76) **		22	0.47(0.22,0.99) *		33	1.32(0.74,2.35)	
dominant	**A/A**	52	Reference	**0.052**	63	Reference	0.9	71	Reference	0.99
	**Any T**	128	0.67(0.44, 1.01) .		99	0.97(0.64,1.49)		143	1.00(0.67,1.48)	
recessive	**Any A**	144	Reference	**0.006**	140	Reference	**0.017**	181	Reference	0.23
	**T/T**	36	0.52(0.33,0.83) **		22	0.42(0.21,0.85) *		33	1.37(0.81,2.32)	
**rs13334970 (*SRL*)** codominat	**G/G**	101	Reference	**0.016**	87	Reference	**0.062**	84	Reference	**0.058**
	**G/A**	63	1.34 (0.91, 1.96)		61	1.18(0.77,1.82)		101	1.54(0.99,2.38) .	
	**A/A**	16	2.54(1.28,5.03) **		12	2.69(1.18,6.13) *		30	0.87(0.47,1.61)	
dominant	**G/G**	101	Reference	**0.033**	87	Reference	0.232	84	Reference	0.18
	**Any A**	79	1.48(1.03,2.11) *		73	1.28(0.85,1.94)		131	1.33(0.88,2.01)	
recessive	**Any G**	164	Reference	**0.014**	148	Reference	**0.026**	185	Reference	0.18
	**A/A**	16	2.33(1.19,4.57) *		12	2.48(1.12,5.50) *		30	0.68(0.39,1.19)	
		**ANGIOPREDICT *KRAS* wt**			**MAVERICC *KRAS* wt**			**TRIBE *KRAS* wt**		
**rs12150220 (*NLRP1*)** codominant	**A/A**	22	Reference	**0.027**	36	Reference	**0.019**	23	Reference	0.67
	**A/T**	50	1.32(0.69,2.54)		42	1.55(0.90,2.68)		50	0.76(0.41,1.40)	
	**T/T**	14	0.46(0.18,1.16) .		8	0.28(0.08,0.98) *		15	0.77(0.31,1.92)	
dominant	**A/A**	22	Reference	0.74	36	Reference	0.62	23	Reference	0.38
	**Any T**	64	1.12(1.58-2.17)		50	1.13(0.67,1.93)		65	0.76(0.42,1.39)	
recessive	**Any A**	72	Reference	**0.019**	78	Reference	**0.02**	73	Reference	0.91
	**T/T**	14	0.41(0.19,0.86) *		8	0.24(0.07,0.80) *		15	0.95(0.44,2.08)	
**rs13334970 (*SRL*)** codominant	**G/G**	45	Reference	**0.06**	43	Reference	**0.025**	37	Reference	0.38
	**G/A**	33	1.23(0.71,2.15)		36	1.41(0.80,2.49)		41	1.19(0.62,2.32)	
	**A/A**	8	4.65(1.29-16.84) *		6	4.04(1.46,11.16) **		10	2.15(0.79,5.85)	
dominant	**G/G**	45	Reference	0.26	43	Reference	0.11	37	Reference	0.32
	**Any A**	41	1.37(0.79,2.35)		42	1.56(0.90,2.69)		51	1.36(0.74,2.50)	
recessive	**Any G**	78	Reference	**0.025**	79	Reference	**0.014**	78	Reference	0.15
	**A/A**	8	4.31(1.20,15.54) *		6	3.37(1.28,8.82) *		10	2.02(0.77,5.30)	

Supplementary Table S3. Replication results for *NLRP1* rs12150220 and *SRL* rs13334970 in two independent cohorts (MAVERICC and TRIBE) in the *KRAS* mutant patients. HRs, the respective 95% confidence intervals (CI) and the across-genotype Wald test p-values are presented under codominant, dominant and recessive models, using multivariable Cox proportional hazard regression models adjusted for available clinical covariates. For each HR specifically, the order of significance of the HR being different from 1 is given by stars (giving the order of magnitude of the probability *p* that the model coefficient for the indicated genotype could be zero) with **.** (dot) 0.05<*p*<0.1; * 0.01≤p≤0.05; ** *p* of order 0.001; *** *p* of order 0.0001 and less. No adjusting according to type of treatment and *BRAF* status for APD *KRAS* mutant patients, because all these patients are were treated with a flouropyrimidine backbone and all *BRAF* codon 600 V/E were wild type.

		**ANGIOPREDICT *KRAS* mut** *Progression-free Survival*			**MAVERICC *KRAS* mut** *Progression-free Survival*			**TRIBE *KRAS* mut** *Progression-free Survival*		
SNPs	*Genotype*	*n*	*Adjusted HR (95%CI)*	*Wald test p-val*	*n*	*Adjusted HR (95%CI)*	*Wald test p-val*	*n*	*Adjusted HR (95%CI)*	*Wald test p-val*
**rs12150220 (*NLRP1*)** codominant	**A/A**	17	Reference	**0.087**	18	Reference	0.29	34	Reference	**0.023**
	**A/T**	29	0.48(0.23,1.02) **.**		27	0.77(0.35,1.70)		45	1.34(0.71,2.52)	
	**T/T**	14	0.39(0.16,0.99) *		11	0.44(0.16,1.23)		10	3.61(1.41,9.24) **	
dominant	**A/A**	17	Reference	**0.03**	18	Reference	0.25	34	Reference	0.22
	**Any T**	43	0.45(0.22,0.93) *		38	0.65(0.31,1.36)		55	1.47(0.80,2.72)	
recessive	**Any A**	46	Reference	0.3	45	Reference	0.16	79	Reference	**0.009**
	**T/T**	14	0.66(0.31,1.43)		11	0.51(0.21,1.29)		10	2.89(1.3,6.43) **	
**rs13334970 (*SRL*)** codominant	**G/G**	36	Reference	0.23	31	Reference	0.66	31	Reference	**0.013**
	**G/A**	22	1.62(0.86,3.05)		21	0.77(0.38,1.59)		44	1.79(0.91,3.54) **.**	
	**A/A**	2	2.94(0.44,19.3)		4	1.39(0.31,6.32)		15	0.62(0.26,1.51)	
dominant	**G/G**	36	Reference	0.11	31	Reference	0.58	31	Reference	0.41
	**Any A**	24	1.66(0.89-3.1)		25	0.82(0.41,1.64)		59	1.32(0.68,2.54)	
recessive	**Any G**	58	Reference	0.39	52	Reference	0.55	75	Reference	**0.019**
	**A/A**	2	2.23(0.35-14)		4	1.57(0.36-6.86)		15	0.41(0.20,0.87) *	

Supplementary Table S4. *STPB* rs229592 in the APD, MAVERICC and TRIBE cohorts in the *KRAS* wt patients. HRs, the respective 95% confidence intervals (CI) and the across-genotype Wald test p-values are presented under codominant, dominant and recessive models, using multivariable Cox proportional hazards models adjusted for available (see Materials and Methods) covariates. For each HR specifically, the order of significance of the HR being different from 1 is given by stars (giving the order of magnitude of the probability *p* that the model coefficient for the indicated genotype could be zero) with **.**(dot) 0.05<*p*<0.1; * 0.01≤p≤0.05; ** *p* of order 0.001; *** *p* of order 0.0001 and less.

		**ANGIOPREDICT *KRAS* wt** *Progression-free Survival*			**MAVERICC *KRAS* wt** *Progression-free Survival*			**TRIBE *KRAS* wt** *Progression-free Survival*		
SNPs	*Genotype*	*n*	*Adjusted HR (95%CI)*	*Wald test p-val*	*n*	*Adjusted HR (95%CI)*	*Wald test p-val*	*n*	*Adjusted HR (95%CI)*	*Wald test p-val*
**rs229592 (*SPTB*)**	**A/A**	43	Reference	**0.003**	38	Reference	0.7	48	Reference	**0.005**
	**A/G**	36	1.81(1.03,3.21) *****		26	0.94(0.50,1.78)		22	1.06(0.52,2.15)	
codominant	**G/G**	7	5.25(1.94,14.23) ******		11	0.69(0.29,1.65)		3	10.31(2.51,42.3) ******	
	**A/A**	43	Reference	**0.013**	38	Reference	0.61	48	Reference	0.36
dominant	**Any G**	43	2.00(1.15,3.46) *****		37	0.86(0.48,1.54)		25	1.36(0.71,2.63)	
	**Any A**	79	Reference	**0.006**	64	Reference	0.41	70	Reference	**0.001**
recessive	**G/G**	7	3.73(1.47-9.46) ******		11	0.71(0.31,1.61)		3	10.26(2.51,42.00) ******	

Supplementary Table S5. Replication results for the combination of *NLRP1* rs12150220 and *SRL* rs13334970 in APD, the two independent cohorts (MAVERICC and TRIBE) and in APD, MAVERICC and TRIBE pooled together, in the overall (upper row) and *KRAS* wt (bottom row) patients respectively. HRs, the respective 95% confidence intervals and the across-genotype Wald test p-values are presented, for multivariable Cox proportional hazard regression models adjusted for available (see Methods) covariates. For each HR, the order of significance of the HR being different from 1 is given by stars (giving the order of magnitude of the probability *p* that the model coefficient for the indicated genotype could be zero) with **.** (dot) 0.05<p<0.1; * 0.01≤*p*≤0.05; ** *p* of order 0.001; *** *p* of order 0.0001 or less.

		**ANGIOPREDICT overall** *Progression-free Survival*			**MAVERICC overall** *Progression-free Survival*			**TRIBE overall** *Progression-free Survival*			**The 3 cohorts combined overall** *Progression-free Survival*		
SNPs	*Genotype*	*n*	*Adjusted HR (95%CI), p*	*Wald test p-val*	*n*	*Adjusted HR (95%CI), p*	*Wald test p-val*	*n*	*Adjusted HR (95%CI), p*	*Wald test p-val*	*n*	*Adjusted HR (95%CI), p*	*Wald test p-val*
**rs12150220 (*NLRP1*)**	***NLRP1*: Any A *SRL*: Any G**	132	Reference	**$9*10^{-7}$**	126	Reference	**0.0086**	157	Reference	0.32	415	Reference	**0.03**
**and**	***NLRP1*: Any A *SRL*: AA**	12	8.27(3.29,20.82) *** *p*=0.000007		11	2.21(0.99,4.93) * *p*=0.05		24	0.75(0.41,1.37) *p*=0.35		47	1.37 (0.97,1.94) **.** *p*=0.07	
**rs13334970 (*SRL*)**	**NLRP1: TT SRL: Any**	36	0.52(0.33,0.83) ** *p*=0.006		22	0.45 (0.22, 0.91) * *p*=0.026		33	1.31(0.77, 2.23) *p*=0.32		90	0.8(0.61,1.04) **.** *p*=0.09	
		**ANGIOPREDICT *KRAS* wt** *Progression-free Survival*			**MAVERICC *KRAS* wt** *Progression-free Survival*			**TRIBE *KRAS* wt** *Progression-free Survival*			**The 3 cohorts combined *KRAS* wt** *Progression-free Survival*		
**rs12150220 (*NLRP1*)**	***NLRP1*: Any A *SRL*: Any G**	62	Reference	**0.005**	70	Reference	**0.0057**	67	Reference	**0.0021**	201	Reference	**0.000001**
**and**	***NLRP1*: Any A *SRL*: AA**	5	4.44(1.23,16.13) * *p*=0.02		6	3.02 (1.15,7.95) * *p*=0.025		6	7.75 (2.47,24.36) *** *p*=0.00045		20	3.55 (2.09,6.05) *** *p*=0.000003	
**rs13334970 (*SRL*)**	***NLRP1*: TT *SRL*: Any**	19	0.41(0.19,0.86) * *p*=0.02		8	0.25(0.075,0.86) * *p*=0.028		15	0.97 (0.44, 2.14) *p*=0.94		37	0.68 (0.44, 1.02) **.** *p*=0.06	
